# Wheelchair Propulsion Biomechanics in Junior Basketball Players: A Method for the Evaluation of the Efficacy of a Specific Training Program

**DOI:** 10.1155/2015/275965

**Published:** 2015-10-12

**Authors:** Elena Bergamini, Francesca Morelli, Flavia Marchetti, Giuseppe Vannozzi, Lorenzo Polidori, Francesco Paradisi, Marco Traballesi, Aurelio Cappozzo, Anna Sofia Delussu

**Affiliations:** ^1^Interuniversity Centre of Bioengineering of the Human Neuromusculoskeletal System, Department of Movement, Human and Health Sciences, University of Rome “Foro Italico”, Piazza Lauro de Bosis 15, 00135 Rome, Italy; ^2^Santa Lucia Foundation, Scientific Institute for Research, Hospitalization and Healthcare, Via Ardeatina 306, 00179 Rome, Italy; ^3^Department of Physiology and Pharmacology “Vittorio Erspamer”, Sapienza University of Rome, Piazzale Aldo Moro 5, 00185 Rome, Italy

## Abstract

As participation in wheelchair sports increases, the need of quantitative assessment of biomechanical performance indicators and of sports- and population-specific training protocols has become central. The present study focuses on junior wheelchair basketball and aims at (i) proposing a method to identify biomechanical performance indicators of wheelchair propulsion using an instrumented in-field test and (ii) developing a training program specific for the considered population and assessing its efficacy using the proposed method. Twelve athletes (10 M, 2 F, age = 17.1 ± 2.7 years, years of practice = 4.5 ± 1.8) equipped with wheelchair- and wrist-mounted inertial sensors performed a 20-metre sprint test. Biomechanical parameters related to propulsion timing, progression force, and coordination were estimated from the measured accelerations and used in a regression model where the time to complete the test was set as dependent variable. Force- and coordination-related parameters accounted for 80% of the dependent variable variance. Based on these results, a training program was designed and administered for three months to six of the athletes (the others acting as control group). The biomechanical indicators proved to be effective in providing additional information about the wheelchair propulsion technique with respect to the final test outcome and demonstrated the efficacy of the developed program.

## 1. Introduction

The benefits of practicing sports and physical activities for individuals with disabilities are generally accepted and concern psychosocial health and functional ability as well as general quality of life [1]. This is particularly the case for young people who, through sports practice, can positively reinterpret their role following a disabling injury and regain self-esteem as well as social integration [2, 3]. Participation in sports activities for young people with disability has remarkably increased over the past years and wheelchair basketball, in particular, has become one of the most popular disciplines among wheelchair-based sports [4].

As a consequence of the increase in participation, competitiveness has risen and the development of training protocols which are specific to the discipline and to the population has become more and more important. In this respect, quantitative biomechanical assessment of performance- and injury-related parameters provides information related not only to the overall outcome (product) of the analysed motor task, but also to the way this task is performed (process).

Extensive literature exists which analyzes biomechanical aspects of wheelchair basketball. In particular, the following areas have been widely investigated: wheelchair propulsion technique [5, 6]; the influence on propulsion mechanical efficiency of different parameters such as push frequency [7], push synchrony [7, 8], push symmetry [9], upper limb kinematics [10], and forces exchanged between the hands and the wheelchair hand rims [11]; the optimal release conditions and corresponding arm movement patterns in the free throw [12]; and the optimisation of the wheelchair configuration and ergonomics [13]. All these studies focused on adult wheelchair athletes and although it has been suggested that differences exist in the biomechanics of wheelchair propulsion between adults and children [14], no information is available about junior wheelchair basketball players. In addition, most of the above-mentioned studies were performed in a laboratory setting, using either an ergometer or a treadmill. However, the laboratory-based approach lacks providing a valid representation of the kinematics and kinetics characterising over-ground propulsion [15]. Therefore, in order to supply coaches with the most meaningful information, it is crucial to develop methods and tools able to provide accurate and reliable data in realistic field-based environments [6, 16].

To this aim, wearable inertial measurement units (IMUs) have been proposed as a valid alternative to traditional laboratory-based instruments, allowing for in-the-field performance monitoring without neither constraining athletes' movements nor significantly modifying the original wheelchair configuration. Few studies focused on the use of IMUs to assess biomechanical parameters in wheelchair sports, such as speed [17], wheelchair displacement and orientation [18], and upper body kinematics [19]. However, IMUs have never been used to characterise wheelchair propulsion in junior wheelchair basketball players nor have been flanked to traditional, or* ad hoc* developed, training programs adopted by wheelchair sports coaches.

Concerning training methodologies, only few studies focused on the development and assessment of training programs specific for wheelchair users. One study [20] reported that strengthening, stretching, and aerobic exercises improved propulsion biomechanical economy in terms of both kinematic and kinetic parameters without increasing shoulder and elbow stresses. The same study, however, included only adult wheelchair users without any previous sports experience. In another study [21], the effects of eccentric exercises on muscle soreness and on shooting percentages were investigated in wheelchair basketball players, and it was proven that muscle soreness induced by eccentric training negatively affects the upper limbs motor performance, at least 48 hours after exercise. Again, only adult athletes were included in the work. Only one study [22] focused on paediatric wheelchair users (ranging in age from 4 to 16 years) and reported that, after 8 weeks of resistance training, the performance of the 12-minute test significantly improved. The participants of the study, however, did not have any previous sports experience. Therefore, the conclusions of the above-mentioned works can hardly be extended to the field of junior wheelchair basketball. Nevertheless, the need to identify which critical factors should be taken into account in the development of training programs that are specific for the discipline and, particularly, the target population has been widely acknowledged [2, 3].

In this framework, the purpose of the present study was twofold: (i) to propose a method based on inertial sensing technologies aimed at obtaining a set of biomechanical parameters able to provide performance-related information about in-the-field wheelchair propulsion and (ii) to develop a discipline- and population-specific training program for junior wheelchair basketball athletes and to assess its efficacy using the proposed biomechanical parameters.

## 2. Methods

### 2.1. Participants

Twelve junior wheelchair basketball players (Table 1) were included in the study after having provided written informed consent. The participants were recruited from the Santa Lucia Foundation junior wheelchair basketball team and had at least two years of previous wheelchair basketball experience. Medical examination identified four athletes with paraplegia, three with myelomeningocele, two with poliomyelitis, one with spastic diplegia, one with a below-knee amputation, and one with a knee arthroprosthesis due to bone cancer. All participants were right-handed. Criterion for exclusion was presence of any medical contraindications which could prevent the athletes from regularly attending the training sessions over the season. The protocol of the study was approved by the Ethics Committee of the Santa Lucia Foundation.

To assess the efficacy of the proposed training program, participants were randomly divided into an experimental (EG) (*N* = 6) and a control group (CG) (*N* = 6). The homogeneity of the two groups in terms of anthropometrical characteristics (age, mass, and stature), functional classification score (*FCS*), and years of wheelchair basketball practice was assessed by means of an unpaired* t*-test, after having verified the normality distribution of the data. No significant difference was found (*p* > 0.05) between the two groups for each tested variable.

### 2.2. Study Design

Three experimental sessions were carried out, during which an instrumented 20-metre sprint test was performed. The first session (ES1) was performed in October, at the beginning of the training season, and was used to fulfil the first aim of the study (i.e., the biomechanical characterisation). The results obtained at this stage were used as a guidance to design a discipline- and population-specific training program (see Section 2.5), which was administered only to EG for three months, during the second half of the competitive season (from February to April). Within the same period, both CG and EG underwent the standard training usually proposed by the team coach. This training was administered twice a week, lasted about 90 minutes, and included exercises aimed at improving all aspects determining wheelchair basketball performances, that is, wheelchair propulsion, wheelchair and ball handling, wheelchair manoeuvrability, and shot and pass accuracy. To assess the efficacy of the proposed discipline- and population-specific training program, CG and EG were tested before and after its administration. Specifically, a second experimental session (ES2) was performed in January and was considered as a baseline, whereas a third and final experimental session (ES3) was carried out in May, at the end of the three-month administration period.

The three experimental sessions, as well as the specific and the standard training program, were performed on the basketball court of the Santa Lucia Foundation and each athlete used the wheelchair commonly adopted during both training practice and competition. The same experimental protocol was followed for all the three sessions, as described in the following section.

### 2.3. Experimental Protocol and Instrumentation

**Figure 1:**
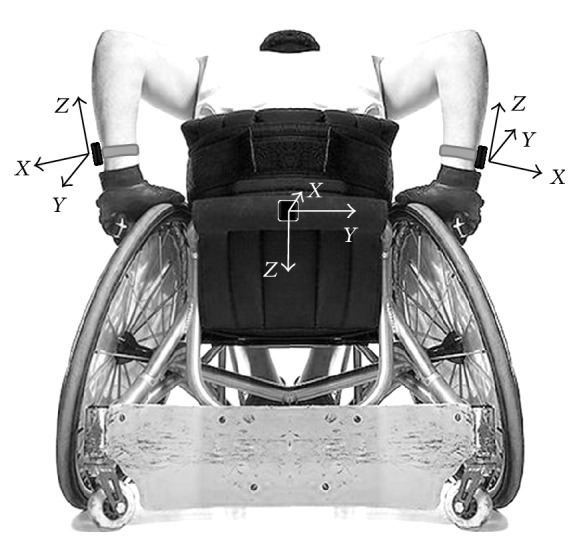
Wheelchair- and wrist-mounted IMUs with the relevant systems of reference.

During each experimental session, each athlete was equipped with three wearable IMUs (Opal, APDM Inc., Portland, Oregon, USA). These devices embed three-axial accelerometers (±6 g of full-range scale, 128 samples/s) providing the components of the vector sum of gravitational and inertial linear accelerations along the axes of a coordinate system fixed with the unit. Two IMUs were fixed on the right and left wrists using elastic bands, while the third unit was securely attached to the backrest of the wheelchair using double-sided tape (Figure 1).

Each athlete performed a 20-metre sprint test (20 mS). This test, focusing in particular on start-up and steady state velocities, was selected within those proposed for adult wheelchair basketball players [23] and then validated in terms of reliability for junior athletes [24]. It includes crucial factors in wheelchair basketball performance, such as starting and sprinting, which involve both strength capacity and coordination skills [23, 25]. Athletes started from a static position with the front wheels behind the start line and, after the start signal, pushed themselves for 20 metres as fast as possible. Time was manually recorded using a digital stopwatch (*t*_20 mS_). The test was performed twice and the trial corresponding to the athletes' shortest time was further considered.

In addition, before the beginning of the competitive season, each subject was medically examined and the peak power output (*PO*) of the upper arms was obtained using an arm crank ergometer (LODE, Groningen, Netherlands). The test protocol was defined according to previous performances obtained by each athlete on the same arm crank ergometer and following the indications of published studies on wheelchair athletes [26, 27]. The initial workload was set to 5 W for the athletes with a* FCS* equal or inferior to 2.5, whereas it was set to 10 W for those athletes with a score higher than 2.5. The workload was increased, every minute thereafter, by 5 or 10 W, according to the* FCS* of each athlete. Participants were instructed to maintain a cranking rate between 60 and 70 revolutions per minute. Testing was terminated when the subject was unable to maintain the required cranking rate or upon the athlete's volitional fatigue (about 10–12 minutes after the beginning of the test). The normalised* PO* with respect to each athlete's mass was hereafter considered as an indicator of the subject's aerobic physical capacity.

### 2.4. IMU Data Processing

To remove random noise, the measured accelerations were low-pass-filtered with a cut-off frequency of 12 Hz using a 4th-order zero-lag Butterworth filter [28]. The calibration of the accelerometers was verified by performing* ad hoc* data collection and following the procedure described in [29]. The inclination with respect to gravity of the IMU located on the wheelchair backrest was computed during the stationary phase (which lasted about five seconds) at the beginning of each trial using IMU data and following the pitch-roll-yaw rotation sequence [29]. The IMU measurements were thus expressed, through a rigid transformation, in a local reference frame having one axis aligned vertically and one aligned with the progression direction. As the IMU was rigidly attached to the wheelchair and no wheelies occurred during the 20 mS, it can be assumed that the above-mentioned system of reference maintained a constant orientation throughout the 20 mS except for rotations occurring about the vertical axis.

**Figure 2:**
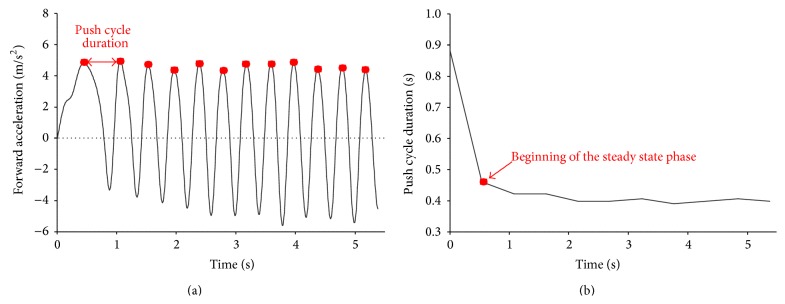
(a) Forward component of the acceleration measured by the wheelchair IMU. Dots indicate relative maxima. The duration of a cycle is also indicated with a double arrow. (b) Trend of the push cycle duration over time. The beginning of the steady state phase is indicated.

The forward component of the acceleration measured by the wheelchair IMU was then used to identify the beginning of the steady state phase. To this aim, the signals were further low-pass-filtered with a cut-off frequency of 4 Hz and relative maxima were detected on the curve (Figure 2(a)). The time duration between two consecutive peaks was calculated and associated with the push cycle duration. The beginning of the steady state phase was set as the instant of time where a rapid change in the time duration slope occurred (Figure 2(b)).

Based on previous literature [9, 10, 30] and on the team coach's suggestions, for each session/subject/trial, the following parameters were estimated from the acceleration signals during the steady state phase.

*Timing.* The push cycle duration (Δ*t*) was defined as the average of the period of time from two consecutive maxima identified on the forward component of the acceleration measured by the IMU located on the backrest of the wheelchair. The push cycle frequency (*f*) was defined as the number of push cycles per minute. 

*Progression Force.* The progression force (*F*_*p*_) was defined as the product of *m* times *a*_*p*_, where *m* is the total mass obtained as the sum of the wheelchair, IMU, and athlete's masses and *a*_*p*_ is the peak value of the forward component of the acceleration measured by the IMU located on the wheelchair. This parameter, which was obtained for each push cycle, represents an estimation of the anteroposterior component of the force applied to the centre of mass of the whole system composed by wheelchair, athlete, and IMU. The average of the *F*_*p*_ parameter over the steady state phase of each trial was calculated and considered for the statistical analysis. 

*Bilateral Symmetry.* A parameter associated with the symmetry (*sym*) between the dominant arm and nondominant arm in pushing the wheelchair was computed for each push cycle according to [30] and considering the peak acceleration measured by the IMUs located on the wrists

(1) $$\begin{array}{c} sym=\frac{a_{p\text{\_dom}}}{\left(a_{p\text{\_dom}}+a_{p\text{\_nondom}}\right)}\cdot 100, \end{array}$$

where *a*_*p*_dom_ and *a*_*p*_nondom_ correspond to the peaks of the acceleration magnitude as measured by the IMUs positioned on the wrist of the dominant and nondominant arms, respectively. This parameter was calculated to investigate whether the dominant and nondominant arm wrists presented similar peak accelerations. A value ranging between 45% and 55% indicates good symmetry, whereas a value lower than 45% or higher than 55% reflects greater accelerations for the nondominant or dominant hand rim, respectively [30]. The average of the* sym* parameter over the steady state phase was calculated and hereafter considered. 

*Intercycle Variability.* The intercycle variability was assessed by computing the coefficient of variation (*CV*) of Δ*t*, *F*_*p*_, and* sym* over all consecutive push cycles of the steady state phase. The mean and standard deviation (SD) of the above listed parameters were calculated and the relative coefficients of variation were obtained as follows:

(2) $$\begin{array}{c} {\text{CV}}_{\text{parameter}}=\left(\;\frac{{\text{SD}}_{\text{parameter}}}{{\text{mean}}_{\text{parameter}}}\;\right)\cdot 100. \end{array}$$

This index is considered as an indicator of the push-to-push movement consistency and low values of the* CV* have been associated with the effective execution of repetitive movement patterns [10, 31], related to task specific coordinative skills [32].

### 2.5. Training Program Definition and Administration

Based on the results of the above-mentioned instrumented 20 mS and on literature evidence related to the demands of wheelchair basketball athletes [20, 23, 33], a training program specific for the population included in the study was developed. This program focused on both strength and coordination training.

Strength exercises involved the following muscle groups: biceps, triceps, middle trapezius, and shoulder abductors and adductors [4]. Due to the presence of athletes affected by spasticity, exercises included limited repetitions (three sets of 15–20 repetitions) [34] and were performed using elastic bands and following the guidelines proposed in the literature [35, 36]. Resistance was modulated by shortening or doubling the elastic bands. All strengthening exercises were performed within the subjects' tolerance and stopped if pain or discomfort was reported.

Coordination exercises focused on the sport specific components of coordination skills and, in particular, on spatial orientation, kinaesthetic differentiation, reaction, adaptation, combination, and rhythm [37]. The intensity of the exercises was increased over the administration time span by using balls of different masses and dimensions (tennis, medicine, soft, and basketball), by combining (limiting) visual or auditive stimuli to specific body movements stressing the cognitive component, and by changing the rhythm of the exercise [38].

Following the guidelines proposed by Faigenbaum et al. [35], the training program was administered to the six athletes of the EG twice a week for three months (12 weeks). Each session lasted 30–35 minutes and was divided into three parts: warm-up, conditioning phase, and cooldown (including stretching and breathing exercises). Within each month, the first and the third weeks were dedicated to coordination exercises whereas the second and the fourth weeks were focused on strength training.

### 2.6. Statistical Analysis

The statistical analysis was performed using the IBM SPSS Statistics software (IBM Corp., Armonk, NY, USA). The alpha level of significance was set to 0.05 for all statistical tests.

#### 2.6.1. Biomechanical Characterisation

For each subject and each trial, the normal distribution of the IMU-based estimated parameters was verified using the Shapiro-Wilk test of normality. As all parameters were not normally distributed, the Spearman (*ρ*) correlation coefficient was used to explore the relationship between the 20 mS performance and each estimated parameter. In addition, to investigate which parameter, among those estimated, could be used to predict the value of *t*_20 mS_, a linear stepwise multiple regression analysis was performed using *t*_20 mS_ as dependent variable and Δ*t*,* f*, *F*_*p*_,* sym*, *CV*_Δ*t*_, *CV*_*F*_*p*__, *CV*_*sym*_, and* FCS* and* PO* as independent variables.

#### 2.6.2. Training Program Assessment

For both ES2 and ES3, each subject, and each trial, the normal distribution of the IMU-based estimated parameters was verified using the Shapiro-Wilk test of normality. As all parameters were not normally distributed, a Wilcoxon signed-rank test was performed to investigate whether significant differences existed between ES2 and ES3 sessions, for both EG and CG. In addition, Mann-Whitney* U* test was carried out to assess whether significant differences existed between EG and CG, for both ES2 and ES3.

## 3. Results

### 3.1. Biomechanical Characterisation

Descriptive statistics of *t*_20 mS_,* FCS*, and* PO* and of all the parameters estimated from the IMU data during ES1 are presented in Table 2. Spearman correlation coefficients between *t*_20 mS_ and each above-mentioned parameter are also reported, together with the relevant *p* value. Strong correlations were obtained for the* FCS*,* PO*, Δ*t*,* f*, and* sym*. Moderate correlations were found for all the coefficients of variation (*CV*_Δ*t*_, *CV*_*sym*_, and *CV*_*F*_*p*__), and a weak negative correlation was obtained for *F*_*p*_. All the reported correlations were statistically significant.

The results of the multiple regression analysis are reported in Table 3. Four predictors were included in the model:* PO*, *CV*_*sym*_, *CV*_Δ*t*_, and* FCS* (*R* ^2^ = 0.826, *R* ^2^_Adjusted_ = 0.818, standard error = 0.484, F(4,7) = 102.205,* p* < 0.001). All of them significantly contributed to predicting values of *t*_20 mS_ and accounted for more than 80% of *t*_20 mS_ variance. Both* PO* and* FCS* had significant negative regression scores, while *CV*_*sym*_ and *CV*_Δ*t*_ had significant positive scores.

### 3.2. Training Program Assessment

The mean and SD of *t*_20 mS_ and of all the IMU-based estimated parameters, together with the results of the comparison between ES2 and ES3 for both the CG and the EG, are reported in Table 4. No significant difference between ES2 and ES3 was found for the CG. Conversely, for the EG, significant differences were obtained for Δ*t*,* f*, *F*_*p*_, and* sym* when comparing those parameters before and after the administration of the training program. No significant difference was found between the two groups, for both ES2 and ES3.

## 4. Discussion

This study presents a method for the quantitative characterisation of a wheelchair basketball field test (20-metre sprint test) based on the use of inertial measurement units (IMUs). A list of biomechanical parameters was defined as performance indicators, which allowed for the development of a sports- and population-specific training program. The method was applied to a team of junior wheelchair basketball athletes and was used to assess the efficacy of the developed training program which was administered for three months.

### 4.1. Biomechanical Characterisation

The proposed instrumented 20-metre sprint test (20 mS) allowed extracting a list of biomechanical indices associated with the performance of the test (*t*_20 mS_). Differently from common performance scores based on the overall outcome (product) of field tests, the estimated biomechanical indices allowed obtaining additional information related to the way this outcome was obtained (process). In particular, from IMU data, parameters were estimated related to the propulsion timing, the progression force that accelerates the wheelchair, the symmetry between the right and left arms in pushing the wheelchair, and the intercycle variability of these parameters.

The results about *t*_20 mS_ are in general agreement with the existing literature, whereas the observed discrepancies are mainly attributed to the different participants' age [24, 39] or to the slightly different testing protocol, which, in other studies, involves the use of the ball during wheelchair propulsion [25]. Similarly, the results about the timing parameters (Δ*t* and* f*) confirm previous findings [8, 40]. Further comparisons with other studies can be hardly performed, as the testing protocols rarely include velocity above 2 m/s [10, 11].

In terms of aerobic power, the present results are in agreement with previous findings about elite wheelchair athletes [27, 41]. According to the existing literature [27], a strong correlation exists between the maximal oxygen consumption VO_2max_ and* PO*. Thus, an estimation of the VO_2max_ can be obtained from the* PO* results, so as to have an overall picture of the athletes' aerobic physical fitness. For the participants of the present study, mean values of predicted VO_2max_ were about 1.7 l/min for female and 2.1 l/min for male athletes. These values are comparable with those reported in [41], where elite wheelchair basketball athletes were considered, indicating that the tested sample had a good level of aerobic physical fitness.

The results of the correlation analysis provide interesting insights into the importance of the biomechanical characterisation of the wheelchair propulsion during the 20 mS. All parameters display a statistically significant relationship with the performance index, *t*_20 mS_ (correlation coefficients ranging from 0.24 to 0.87), indicating that the estimated indicators not only provide information about the athletes' wheelchair propulsion technique, but also are descriptive of the overall test performance.

In particular, the peak power output (*PO*), the* FCS*, both timing parameters (Δ*t* and* f*), and the bilateral symmetry index (*sym*) strongly correlate with *t*_20 mS_. These results, which confirm the findings of previous studies [8, 10], reveal that better performances are related to the ability of the athletes to generate high power with the upper arms as well as to increase the push frequency (thus decreasing the push cycle duration) and the push symmetry between the right and left arms during propulsion. In particular, as far as the push symmetry is concerned, four main aspects emerge from the existing literature: (i) symmetrical and synchronous pushing modes are associated with greater wheelchair velocity and push power [8]; (ii) the presence of upper-extremity asymmetry when pushing the wheelchair may contribute to the development of injury [9]; (iii) a close relationship exists between upper arms coordination and both technical efficiency and injury prevention [33]; (iv) symmetry indices are often used to describe upper arm coordination in different sport activities [42]. The results of the present work, therefore, confirm the importance of the push symmetry as a valuable performance- and injury-related indicator.

Moderate positive correlations were obtained for all the computed coefficients of variation (*CV*), showing that the lower the intercycle variability, the lower the *t*_20 mS_. This is in agreement with the idea that a high level of expertise results in the capacity to reproduce a movement like an automatism [43] and that low values of* CV* are associated with the effective execution of repetitive movement patterns [10, 31]. This does not imply that expert athletes are able to reproduce identical movement patterns but rather that they are capable of picking up several sources of information (visual, haptic, and acoustic) to perform different movements and to use the so-called coexisting modes of coordination [32] to achieve consistent and effective functional performance outcomes [43].

A significant negative relationship between *t*_20 mS_ and the peak progression force (*F*_*p*_) was found, although less evident than that of other parameters. In particular, this correlation suggests that, as expected, the higher the force that accelerates the wheelchair, the better the test outcome. The relationship between the wheelchair propulsion efficiency and the force applied by each arm on the hand rim, as well as the symmetrical/asymmetrical distribution of this force, is well established in the literature. In particular, the greater the force is (or the power output), the more mechanically efficient the wheelchair propulsion is [10, 20]. The weak correlation between *F*_*p*_ and *t*_20 mS_ found in the present study can be related to the fact that *F*_*p*_ does not coincide with the actual force applied by the athlete on the hand rims, but it is rather an estimation of the anteroposterior component of the force applied to the centre of mass of the whole system composed by wheelchair, athlete, and IMU. Still, this parameter proved to be significantly related to the athletes' performance and can be estimated directly in the field and in real training conditions.

According to the results of the correlation analysis, the regression model identified four significant parameters able to predict *t*_20 mS_, that is,* PO*, *CV*_*sym*_, *CV*_Δ*t*_, and* FCS*. Based on these results, two aspects were selected as targets of the designed discipline- and population-specific training program: (i) strength, here represented by* PO* [10, 20], and (ii) coordination, here represented by *CV*_*sym*_ and *CV*_Δ*t*_ [32]. This is in agreement with the existing literature which identifies wheelchair propulsion dynamics and eye-hand coordination as the two most important constructs for successful wheelchair basketball performance [23, 25].

### 4.2. Training Program Assessment

In the present study, an* ad hoc* training program aimed at improving both strength capacity and coordination skills was developed. On the one hand, no significant difference was found for *t*_20 mS_ after three months of administration, neither for the CG nor for the EG, indicating that the athletes' performance during the 20 mS did not change from ES2 to ES3. This result is in agreement with a previous study [22] which assessed the efficacy of resistance training on six paediatric wheelchair users. In this study, no significant difference was found in the performance of a 50-metre dash test after 8 weeks of training. It should be noticed that, in the present study, the scores of the athletes were similar to those displayed by elite athletes of the same discipline [39] in all three experimental sessions. This aspect, together with the good aerobic physical fitness of the athletes, suggests that there may be limited room for improvement and that three months of program administration could be too short to produce significant changes in the 20 mS final outcome. In addition, the time taken by each subject to complete the test was recorded manually with a digital stopwatch as provided by the established protocol [10, 23–25]. Therefore, the accuracy in measuring the time to complete the test is of the same order as the reaction time typical of thumb movements, that is, about 0.3 s [44], and the detected changes of *t*_20 mS_ are, indeed, within this margin. If actual variations in *t*_20 mS_ take place, they are below the accuracy threshold and, thus, hardly detectable with this instrumentation.

On the other hand, when considering the IMU-based biomechanical parameters related either to strength or coordination, differences between ES2 and ES3 were obtained for the EG only. In particular, significant improvements were displayed for Δ*t*,* f*, *F*_*p*_, and* sym*, showing that EG athletes modified their propulsion technique increasing the push cycle frequency, the force expressed to accelerate their wheelchair, and adopting a more symmetrical pushing mode. In addition, all the* CV* parameters slightly decrease from ES2 to ES3 (*p* > 0.05), suggesting a more effective execution of the repetitive pushing movements, typical of expert athletes [31].

These results indicate that although the administered training program does not influence the final outcome of the 20 mS, it affects the way this outcome is obtained by the athletes. This supports the hypothesis that biomechanical analysis can effectively provide additional performance indicators to the coaches relative to the way specific movements are performed, not limiting the analysis on the final product of the selected motor task.

### 4.3. Limitations of the Study

The investigation included a relatively small sample size, particularly when considering the control and experimental groups separately (i.e., during ES2 and ES3). Also, the study involved participants with different pathologies and variable level of spinal cord lesions. It is plausible that these two aspects play an important role in determining the lack of significant differences between CG and EG after the training program administration. Still, significant differences for a list of biomechanical parameters were identified within each group when comparing the results obtained before and after the administration of the program. It is possible that between-group differences might have been pointed out if a larger cohort of athletes would have been admitted to the program.

Conversely, no statistical difference was detected when comparing the times to complete the 20 mS. This is probably related to the inadequate level of accuracy of the manual digital stopwatch, which could be improved by using photocells or a laser gun. Moreover, it is not excluded that differences in the test score could emerge from a program administration period greater than three months.

In the present study, the progression force was estimated by analysing the acceleration signals measured by the IMU located on the wheelchair. *F*_*p*_ represents, therefore, an estimation of the anteroposterior component of the force applied to the centre of mass of the whole system composed of wheelchair, athlete, and IMU. Although this parameter does not represent the actual force applied by each arm on the wheel hand rim, it proved to be effective in revealing the changes occurring after the training program administration. Furthermore, contrary to traditional experimental protocols which need either to modify the wheelchair with instrumented wheels (increasing the wheelchair mass) or to use a static ergometer, *F*_*p*_ can be estimated directly in the field and in real training conditions by using only one IMU located on the back of the wheelchair.

## 5. Conclusions

This paper fills an existing gap in the field of junior wheelchair basketball. A methodology for the biomechanical assessment of the wheelchair propulsion was developed and a list of biomechanical indices associated with the performance of a 20-metre sprint test was obtained by means of wheelchair- and wrists-mounted inertial measurement units. These indices proved to correlate with the test performance and provide quantitative information about the way athletes obtained such performance. Therefore, they were used to define a sports- and population-specific training program focused on strength and coordination training. The proposed biomechanical methodology was then used to assess the efficacy of the defined training program after three months of administration. The estimated indices were effective in identifying both strength and coordination improvements following the training administration.

Both the biomechanical assessment method and the training program proved to be well perceived by the athletes and to be applicable in training conditions. Special attention, in fact, was paid to the organisational and practical aspects of the experimental protocol and of the program administration. It is worth underlining that the results of the present study were achieved thanks to the effective interaction within the multidisciplinary research group, which allowed addressing and answering the needs of both coaches and physiotherapists, through the complementary expertise of biomechanists, in terms that were valuable to the former professionals.

## Figures and Tables

**Table 1:** Participant characteristics.

Subject	Age [years]	Mass [kg]	Stature [m]	*FCS*	Years of practice	Gender	Group
S1	20	66	1.70	2.5	7	Male	CG
S2	18	48	1.68	3	6	Male	CG
S3	15	42	1.66	2	4	Female	CG
S4	17	61	1.75	0.5	3	Male	CG
S5	12	46	1.45	2	2	Male	CG
S6	17	96	1.78	4.5	3	Male	CG
S7	20	48	1.55	2	7	Male	EG
S8	19	49	1.69	3	7	Male	EG
S9	16	42	1.66	2.5	5	Female	EG
S10	18	74	1.75	0.5	4	Male	EG
S11	13	45	1.45	2	3	Male	EG
S12	20	105	1.90	4.5	3	Male	EG
Descriptive statistics	17.1 ± 2.7	60.2 ± 21.4	1.7 ± 0.1	2.25 ± 1	4.5 ± 1.8		

Characteristics of all participants: descriptive statistics are reported in terms of mean ± standard deviation (SD), except for *FCS* where median ± interquartile range (IQR) is indicated. *FCS*: functional classification score (ranging from 0.5 to 4.5 as proposed by the Italian Paralympic Committee); CG: control group; EG: experimental group.

**Table 2:** Results of the biomechanical characterisation at ES1.

Parameter	Descriptive statistics	Correlation with *t*_20 mS_	
		*ρ*	*p* value
*t*_20 mS_ [s]	6.4 ± 1.1		
*FCS*	2.25 ± 1	−0.675	*p* < 0.001
*PO* [W/kg]	1.46 ± 0.69	−0.870	*p* < 0.001
Δ*t* [s]	0.47 ± 0.06	0.757	*p* < 0.001
*f* [push/min]	113.93 ± 14.86	−0.631	*p* < 0.001
*F*_*p*_ [N]	342.10 ± 240.37	−0.242	*p* < 0.05
*sym* [%]	48.77 ± 3.27	−0.659	*p* < 0.001
*CV*_Δ*t*_ [%]	6.86 ± 6.67	0.548	*p* < 0.001
*CV*_*F*_*p*__ [%]	11.09 ± 6.84	0.448	*p* < 0.001
*CV*_*sym*_ [%]	4.58 ± 3.16	0.520	*p* < 0.001

Descriptive statistics and Spearman correlation coefficients of parameters obtained during ES1. Data are expressed as mean ± SD except for *FCS* reported as median ± IQR. *t*_20 mS_: time to complete the 20-metre sprint test; *FCS*: functional classification score; *PO*: upper arms peak power output; Δ*t*: push cycle duration; *f*: push cycle frequency; *F*_*p*_: peak progression force; *sym*: bilateral symmetry index; *CV*_Δ*t*_: coefficient of variation of the push cycle duration; *CV*_*F*_*p*__: coefficient of variation of the peak progression force; *CV*_*sym*_: coefficient of variation of the bilateral symmetry index.

**Table 3:** Results of the multiple regression model.

Parameter	Unstandardized coefficients		*t*	*p* value	*β* confidence interval (95%)	
	*β*	Std. error			Lower bound	Upper bound
Constant	7.344	0.225	32.593	*p* < 0.001	6.896	7.792
*PO*	−1.021	0.104	−9.799	*p* < 0.001	−1.229	−0.814
*CV*_*sym*_	0.100	0.020	4.998	*p* < 0.001	0.060	0.140
*CV*_Δ*t*_	0.031	0.009	3.634	*p* < 0.001	0.014	0.048
*FCS*	−0.151	0.052	−2.906	*p* < 0.005	−0.254	−0.048

Unstandardized *β* coefficients of the regression equation and relevant statistics (standard error, *t*-test results in terms of *t* and *p* value, 95% confidence interval). Constant: constant parameter; *PO*: upper arms peak power output; *CV*_*sym*_: coefficient of variation of the bilateral symmetry index; *CV*_Δ*t*_: coefficient of variation of the push cycle duration; *FCS*: functional classification score.

**Table 4:** Results of the training program assessment (ES2 and ES3 results).

Parameter	Group	ES2	ES3	Wilcoxon test	
		Mean ± SD	Mean ± SD	*p* value	*Z*
*t*_20 mS_ [s]	CG	6.1 ± 1.2	5.9 ± 1.1	0.248	−1.156
	EG	6.1 ± 1.2	6.2 ± 1.1	0.080	−1.753

Δ*t* [s]	CG	0.44 ± 0.05	0.45 ± 0.08	0.705	−0.378
	EG	0.47 ± 0.09	0.44 ± 0.08	0.027^∗^	−2.207

*f* [push/min]	CG	117.32 ± 14.62	118.30 ± 21.02	0.600	−0.524
	EG	112.65 ± 18.71	119.97 ± 15.74	0.028^∗^	−2.201

*F*_*p*_ [N]	CG	276.56 ± 148.16	289.57 ± 241.16	0.463	−0.734
	EG	330.71 ± 191.10	588.32 ± 312.29	0.028^∗^	−2.201

*sym* [%]	CG	48.91 ± 3.82	47.86 ± 3.82	0.173	−1.363
	EG	47.77 ± 3.56	48.62 ± 3.96	0.046^∗^	−1.992

*CV*_Δ*t*_ [%]	CG	4.36 ± 3.64	6.65 ± 4.81	0.173	−1.363
	EG	5.00 ± 3.38	4.12 ± 4.59	0.345	−0.943

*CV*_*F*_*p*__ [%]	CG	12.00 ± 6.93	15.24 ± 10.47	0.249	−1.153
	EG	12.16 ± 9.92	7.41 ± 3.12	0.249	−1.153

*CV*_*sym*_ [%]	CG	5.57 ± 4.38	4.56 ± 3.41	0.249	−1.153
	EG	4.35 ± 2.74	4.17 ± 2.83	0.917	−0.105

Descriptive statistics and Wilcoxon signed-rank test results (in terms of *p* value and *Z* value) for both the CG and the EG, before (ES2) and after (ES3) the training program administration. Significant differences are indicated with an asterisk. Data are expressed as mean ± SD. *t*_20 mS_: time to complete the 20-metre sprint test; Δ*t*: push cycle duration; *f*: push cycle frequency; *F*_*p*_: peak progression force; *sym*: bilateral symmetry index; *CV*_Δ*t*_: coefficient of variation of the push cycle duration; *CV*_*F*_*p*__: coefficient of variation of the peak progression force; *CV*_*sym*_: coefficient of variation of the bilateral symmetry index.

## Symbols

IMU: Inertial measurement unit
CG: Control group
EG: Experimental group
ES1, ES2, and ES3: First, second, and third experimental sessions, respectively
20 mS: 20-metre sprint test
*FCS*: Functional classification score
*PO*: Upper arms peak power output
*t*_20 mS_: Time to complete the 20-metre sprint test
Δ*t*: Push cycle duration
*f*: Push cycle frequency
*F*_*p*_: Peak progression force
*a*_*p*_: Peak acceleration
*sym*: Bilateral symmetry index
*CV*: Coefficient of variation
SD: Standard deviation
IQR: Interquartile range.
